# 

**Published:** 1861-07

**Authors:** 


LECTURES ON THE DIAGNOSIS OF THE PRINCIPAL FORMS OF PARALYSIS OF THE LOWER EXTREMITIES.—By E. Brown-Séquard, M. D., F. R. S., Fellow of the Royal College of Physicians of London; Hon. Fellow of the Faculty of Physicians and Surgeons, Glasgow; Laureate of the Institute of France, (Academy of Sciences,) Etc., Etc. Philadelphia: J. B. Lippincott & Co. 1861.

COURSE OF LECTURES ON THE PHYSIOLOGY AND PATHOLOGY OF THE CENTRAL NERVOUS SYSTEM.—Delivered at the Royal College of Surgeons of England in May, 1858. By C. E. Brown-Séquard, M. D., F. R. S., Fellow of the Royal College of Physicians of London; Hon. Fellow of the Faculty of Physicians and Surgeons, Glasgow; Laureate of the Institute of France, (Academy of Sciences); Physician to the National Hospital for the Paralyzed and the Epileptic; Ex-Professor of the Institute of Medicine at the Medical College of Virginia, U. S.; Fellow of the Royal Medico-Chirugical Society of London; Ex-Secretary and Vice-President of the Société de Biologié of Paris. Philadelphia: Collins, Printer, 705 Jayne Street. 1860.

A TREATISE ON THE PRACTICE OF MEDICINE.—By EDWIN R. MAXSON, M. D., formerly Lecturer on the Institutes and Practice of Medicine in the Geneva Medical College. Philadelphia: Lindsay & Blackiston. 1861.

We acknowledge the receipt of the above volumes, the first two through S. C. Griggs & Co., 39 and 41 Lake Street, Chicago, and promise suitable reviews in a future number of the EXAMINER.

**N. S. DAVIS, M. D., Editor.---FRANK W. REILLY, M. D., Ass't Editor.**

A MONTHLY JOURNAL,

DEVOTED TO THE

**EDUCATIONAL, SCIENTIFIC, AND PRACTICAL INTERESTS**

OF THE

**MEDICAL PROFESSION.**

The Examiner will be issued during the first week of each month, commenceing with January, 1860. Each number will contain 64 pages of reading matter, the greater part of which will be filled with such contents as will directly aid the practitioner in the daily practical duties of his profession.

To secure this object fully, we shall give, in each number, in addition to ordinary original articles, and selections on practical subjects, a faithful report of many of the more interesting cases presented at the Hospitals and College Cliniques. While aiming, however, to make the Examiner eminently practical, we shall not neglect either the scientific, social, or educational interests of the profession. It will not be the special organ of any one institution, society, or clique; but its columns will be open for well-written articles from any respectable member of the profession, on all topics legitimately within the domain of medical literature, science and education.

Terms, $2.00 per annum, invariably in advance.

